# Complete Genome Sequence of Bovine Adenovirus Type 7 Strain Fukuroi, Isolated from a Cow with Respiratory Disease

**DOI:** 10.1128/MRA.00055-21

**Published:** 2021-03-11

**Authors:** Asuka Kumagai, Sayo Kajikawa, Ayako Miyazaki, Shinichi Hatama

**Affiliations:** a Division of Viral Disease and Epidemiology, National Institute of Animal Health, National Agriculture and Food Research Organization, Tsukuba, Ibaraki, Japan; b Himeji Livestock Hygiene Service Center, Himeji, Hyogo, Japan; KU Leuven

## Abstract

We determined the complete genome sequence of the bovine adenovirus type 7 prototype strain Fukuroi using next-generation sequencing technology. We found that the viral genome is 30,034 bp long and has the shortest inverted terminal repeats among known adenoviruses.

## ANNOUNCEMENT

Bovine adenovirus type 7 (BAdV-7), a member of the *Atadenovirus* genus of the *Adenoviridae* family, was first isolated in 1965 in our laboratory from blood samples from a Holstein cow with respiratory and enteric disease (1–3). BAdV-7 is recognized as one of the most important respiratory and enteric pathogens for the cattle industry (1, 4). Currently, bovine vaccines available in Japan contain BAdV-7 (http://www.kyotobiken.co.jp/en/products/cow.html#respiration). Despite the importance of this virus, its genomic information is limited (5–7). Only 14% of the viral genome, i.e. 2,700 bp, 900 bp, and 623 bp of the hexon, protease, and DNA polymerase genes, respectively (GenBank accession no. AF238232, X53989, and U57335, respectively), is currently available. In this study, we determined the complete genome sequence of the BAdV-7 prototype strain Fukuroi, which was isolated in 1965.

Fukuroi was propagated in bovine embryonic testicle cells as described previously (1). Supernatants were collected 4 days after virus inoculation and centrifuged to remove cellular debris. The stocks were semipurified by discontinuous sucrose density gradient ultracentrifugation and were dissolved in phosphate-buffered saline. DNA was extracted using a QIAamp MinElute virus spin kit (Qiagen) and submitted to Macrogen Japan Co. Ltd. (Tokyo, Japan) for whole-genome sequencing. Briefly, sequencing libraries were constructed using a TruSeq Nano DNA sample preparation kit (Illumina). DNA sequencing was performed with a deep sequencing protocol using a NovaSeq 6000 system (Illumina). A total of 31 million paired-end reads (a total of 4.7 billion bases) with an average length of 151 bp were obtained. The bases with a Phred quality score below 20 were trimmed from every read and assembled with a *de novo* approach using Trimmomatic version 0.36 (http://www.usadellab.org/cms/?page=trimmomatic) and SPAdes version 3.13.0 (http://cab.spbu.ru/software/spades) with default settings. Consequently, a 29,799-bp contig was generated with an average base coverage depth of 4,609×. To make up for the short nucleotide stretches that were lacking at both ends of the genome, 5′ and 3′ adapter ligation (5′-GCCTGATAGCTCACGACTAG-3′), followed by PCR with specific primers (5′-TATTGCCTCAGCAGGAACAC-3′ and 5′-GAATCGTTTCCAATACTGCTTC-3′ for the 5′ and 3′ termini, respectively), and then Sanger sequencing were performed. As a result, the full-length genome sequence of Fukuroi was obtained (30,034 nucleotides with a GC content of 33.56%).

**FIG 1:**
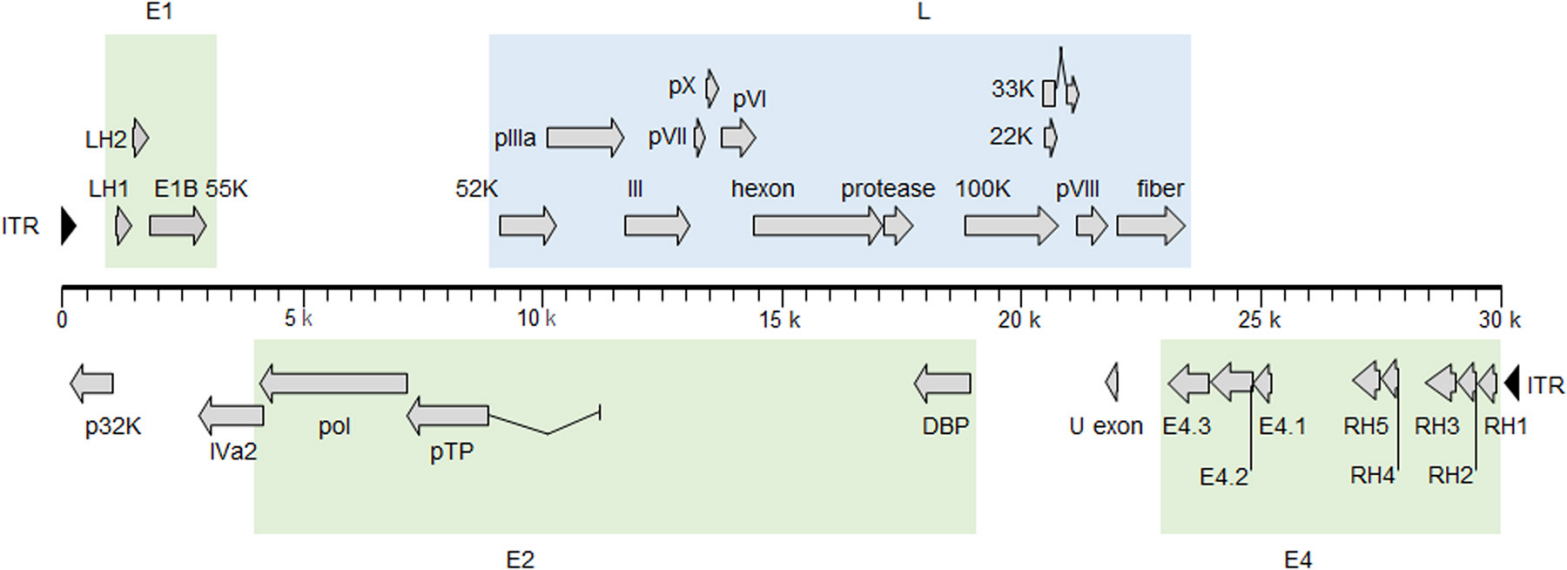
Gene organization in BAdV-7 strain Fukuroi. Predicted protein-coding regions are depicted as gray arrows with the appropriate orientation, and gray rectangles denote protein-coding exons. ITR elements located at both ends of the genome are shown as solid arrowheads. Green and blue boxes indicate early (E1, E2, and E4) and late (L) gene coding regions, respectively.

Genome comparisons demonstrated high nucleotide identity values (from 99.5 to 99.8%) with respect to partial sequences of Fukuroi available in the GenBank database. Putative open reading frames and functions of the translated products were predicted using the DNA Data Bank of Japan (DDBJ) fast annotation and submission tool (https://dfast.nig.ac.jp) (Fig. 1 and Table 1). The inverted terminal repeat (ITR) sequences were 36 bp long. To our knowledge, these are the shortest ITR sequences among the known adenoviruses (8). The whole-genome sequence of Fukuroi will expedite the acquisition of new knowledge on viral evolution, molecular epidemiology of BAdV-7, and vaccination outcomes in Japan.

**TABLE 1:** Description of open reading frames in the genome of BAdV-7 strain Fukuroi

Protein designation	Predicted function	S or NS^*a*^	Length		Genomic (nucleotide) position^*b*^
			No. of base pairs	No. of amino acids	
p32K	Unknown	S	921	306	235–1155 (c-strand)
LH1	Unknown	NS	354	117	1197–1550
LH2	Unknown	NS	375	124	1535–1909
E1B 55K	Large T antigen	NS	1,149	382	1959–3107
IVa2	DNA packing	S	1,095	364	3115–4209 (c-strand)
Pol	DNA polymerase	NS	3,237	1,078	4194–7430 (c-strand)
pTP	Terminal protein	NS	1,788	595	7412–9184, 11910–11924 (c-strand)
52K	DNA packing protein	S	996	331	9203–10198
pIIIa	Minor capsid protein	S	1,713	570	10183–11895
III (penton)	Major capsid protein	S	1,359	452	11937–13295
pVII	Major capsid protein	S	336	111	13337–13672
pX	Minor core protein	S	213	70	13694–13906
pVI	Minor capsid protein	S	636	211	13947–14582
Hexon	Major capsid protein	S	2,787	928	14545–17331
Protease	Protease	S	609	202	17328–17936
DBP	DNA binding protein	NS	1,146	381	17940–19085 (c-strand)
100K	Hexon scaffold protein	S	1,887	628	19105–20991
22k	DNA packing/assembly protein	S	198	65	20858–21055
33k	DNA packing/assembly protein	S	408	135	20858–21035, 21138–21367
pVIII	Minor capsid protein	S	654	217	21399–22052
U exon	Replication center protein	S	165	54	22064–22228 (c-strand)
Fiber	Major capsid protein	S	1,407	468	22236–23642
E4.3	p53 and p73 inhibitor	NS	654	217	23645–24298 (c-strand)
E4.2	p53 and p73 inhibitor	NS	660	219	24298–24957 (c-strand)
E4.1	p53 and p73 inhibitor	NS	432	143	24957–25388 (c-strand)
RH5	Unknown	NS	603	200	27083–27685 (c-strand)
RH4	Unknown	NS	438	145	27688–28125 (c-strand)
RH3	Unknown	NS	483	160	28345–28827 (c-strand)
RH2	Unknown	NS	372	123	28854–29225 (c-strand)
RH1	Unknown	NS	597	198	29330–29926 (c-strand)

a S, structural; NS, nonstructural.

b c-strand, complementary strand.

### Data availability.

The raw read data and the complete genome sequence of Fukuroi have been deposited in the SRA under the accession no. DRR257739 and DDBJ under the accession no. LC597488, respectively.
